# Deformation Control in Mesoscale Micro-Milling of Curved Thin-Walled Structures

**DOI:** 10.3390/ma17205071

**Published:** 2024-10-17

**Authors:** Jie Yi, Xinyao Wang, Yichen Zhu, Xurui Wang, Junfeng Xiang

**Affiliations:** 1 School of Materials Science and Engineering, Shandong Jianzhu University, Jinan 250101, China; yijie18@sdjzu.edu.cn (J.Y.); 19861432095@163.com (X.W.); wxr19961114@163.com (X.W.); 2 Department of Mathematics, University of Manchester, Manchester M13 9PL, UK; yichen.zhu-2@student.manchester.ac.uk; 3 School of Civil Aviation, Northwestern Polytechnical University, Xi’an 710072, China; 4 Innovation Center NPU Chongqing, Northwestern Polytechnical University, Chongqing 401135, China; 5 Yangtze River Delta Research Institute of NPU, Suzhou 215400, China

**Keywords:** mesoscale micro-milling, curved thin-walled structures, deformation control, improved NSGA-III, entropy weight–TOPSIS

## Abstract

The micro-machining scale effect makes it challenging to forecast and control the process parameters of the micro-milling process, which makes the micro-flanking-milling of weak-rigidity micro-thin-walled parts prone to deformation. To determine the critical cutting parameters for chip formation in the micro-milling of curved thin-walled parts at the mesoscale, the strain-softening effect of titanium alloy during high-speed milling and the scale effect of mesoscale cutting were comprehensively considered and a finite element prediction model for curved micro-thin-wall micro-milling was established to determine the critical milling parameters for effective material removal. Based on the determined milling parameters, an experimental design of response surface optimization was carried out. Based on the response surface methodology, a data-driven quantitative model with milling process parameters as design variables and deformation amounts as response variables was established to reveal the influence mechanism of multiple milling process parameters on machining accuracy. Based on the process requirements for deformation control in the micro-milling of curved thin-walled structures, dynamic optimization of the milling process parameters was performed using an improved NSGA-III algorithm to obtain non-dominated solutions. A visual ranking and a determination of the unique solution were conducted using the entropy weight–TOPSIS method. Finally, micro-milling validation experiments were carried out using the optimal parameter combination. The optimal solution for the process parameters of the arc-shaped micro-thin-wall micro-milling of titanium alloy established by the institute provides a relevant reference and guidance for mesoscale arc-shaped thin-wall micro-milling.

## 1. Introduction

TC4 titanium alloy is widely used in aerospace, military, and medical fields due to its excellent combination of mechanical properties, corrosion resistance, and high-temperature stability. However, compared with traditional machining methods, the micro-milling of thin-walled titanium alloy structures at the mesoscale presents unique challenges. The thin-walled features of titanium alloy are highly susceptible to deformation under the influence of milling parameters, resulting in non-uniform wall thickness and difficulties in achieving dimensional accuracy. Given these challenges, optimizing micro-milling parameters for mesoscale thin-walled titanium alloy structures through multi-objective optimization becomes critically important. By reducing machining-induced deformation, the optimization process ensures greater structural integrity and precision in the final part, which is essential for high-performance applications in fields that demand stringent tolerances and reliability.

In recent years, the demand for micro-scale components has gradually increased, making mesoscale manufacturing a hot topic in modern manufacturing technology. Numerous scholars have conducted extensive research on mesoscale cutting. Among the areas studied, the most discussed topics involve the scale effect and the theoretical study of the minimum cutting thickness in micro-cutting. Weule et al. [1] identified the existence of a minimum cutting thickness, conducting cutting experiments with micro-tools to establish the relationship between tool geometry and the minimum cutting thickness. Liu et al. [2], using molecular dynamics principles, proposed a theoretical formula for the factors influencing the minimum cutting thickness under different parameters. Vogler et al. [3] developed a micro-milling force prediction model based on a slip-line model that considered both the minimum cutting thickness and material microstructure. Wu et al. [4] discovered, using strain gradient theory in the cutting of 1045 steel, that flow stress is inversely proportional to the cutting depth. Lucca et al. [5], through cutting experiments on OFHC copper, revealed the scale effect of cutting energy on micro-machining based on comparative analyses of multiple experiments.

High-speed machining allows an excellent surface finish and a good dimensional accuracy in the manufacturing process [6]. Mesoscale-specific phenomena in machining bring about unique theoretical and technological challenges compared with macroscale cutting. It is essential to conduct theoretical and simulation-based studies to understand these characteristics and to reveal the underlying mechanisms during mesoscale cutting [7]. These studies can also provide theoretical support to optimize machining processes. Thepsonthi et al. [8] performed a finite element analysis (FEA) on the micro-milling of titanium alloys, revealing that different material models significantly affect chip formation but have minimal influence on the cutting force. Zhou et al. [9] established a multi-tooth tool cutting simulation model to study the impact of cross-scale machining parameters on the surface morphology of workpieces. Parida et al. [10] used an FEA to develop a two-dimensional cutting simulation model for Inconel 718, analyzing the trend of cutting forces and temperature variations with respect to the tool’s tip radius during mesoscale cutting. Saffarr et al. [11] built a three-dimensional mesoscale milling model for AISI 1045 steel, considering the influence of the tool’s helix angle on the cutting outcomes compared to two-dimensional simulations. Liu et al. [12] used a modified Johnson–Cook constitutive model to establish a two-dimensional orthogonal cutting model for the mesoscale milling of steel rails. Mebrahitom et al. [13] developed a three-dimensional milling FEA model for 6010 aluminum alloy and created a mesoscale cutting force prediction model based on the simulation data. Desai et al. [14] built a two-dimensional mesoscale cutting simulation model for aluminum alloys based on multi-scale plasticity constitutive equations, providing theoretical guidance for practical machining. Mali et al. [15] developed a two-dimensional cutting model for Al7075, using orthogonal experimental simulations to determine how machining parameters influence the cutting force during mesoscale cutting. Young et al. [16] studied the effects of tool parameters on residual stress using an FEA and examined how these stresses impact deformation in thin-walled part machining. Mahnama et al. [17] combined an FEA with chatter dynamics to study mesoscale cutting and explored the process stability under different cutting conditions by examining the interaction between chatter and chip formation. Lotfi et al. [18] used an FEA to predict mesoscale cutting forces and the geometry of chip breakage.

The selection of appropriate milling parameters is crucial to ensure the dimensional accuracy of curved micro-thin-walled titanium alloy parts. To address the nonlinear multi-objective optimization challenges in this process, optimization algorithms are typically employed to derive optimal solutions for multiple objective functions. Rai et al. [19] considered the influence of tool geometry and milling parameters on elastic deformation during thin-walled part milling, developing an FEA model to analyze the relationship between various variables and deformation. Mia et al. [20] employed the response surface methodology (RSM) to establish a mathematical model for specific cutting energy and surface roughness, analyzing the effects of various parameters. Lu et al. [21] used RSM to develop a fiber hardness model for the micro-milling of Inconel 718 and studied the impact of different parameters on microhardness. Li et al. [22] proposed a multi-objective optimization method based on a weighted gray correlation analysis and RSM, applying it to optimize cutting parameters in milling. Zheng et al. [23] integrated a prediction model with a particle swarm optimization (PSO) algorithm to find the optimal parameters to maximize the material removal rate and experimentally validated the prediction model and optimization results. Gianni Campatelli et al. [24] introduced an experimental approach to optimize process parameters, utilizing RSM to analyze the process and develop a model for suitable process parameters. Agwa et al. [25] investigated the effects of various machining processes on the surface quality and applied an algorithm to optimize cutting parameters to improve the machining quality. Zhou et al. [26] addressed energy consumption in cylindrical cutting and optimized machining parameters using a genetic algorithm, minimizing energy consumption during part processing. Hsin et al. [27] constructed a machining model using polynomial networks and employed a simulated annealing optimization algorithm to determine the best cutting parameters. Zain et al. [28] optimized milling parameters using a genetic algorithm to solve the issue of excessive surface roughness in titanium alloy end milling. Thanongsak and Ozel et al. [29] used a multi-objective particle swarm optimization algorithm to optimize cutting parameters, reducing burr formation and surface roughness in the workpieces processed under optimal parameters. Zhou et al. [26] aimed to reduce energy consumption and processing costs, optimizing cylindrical cutting parameters using the NSGA-II genetic algorithm model. Chen et al. [30] considered workpiece deformation and tool vibration factors, and optimized cutting parameters using the PSO algorithm. Paul Tarisai et al. [31] optimized the tool structure and milling parameters using a genetic algorithm to extend the tool life. Shunmugam et al. [32] developed a bi-objective optimization model for milling parameters using a genetic algorithm, aiming to reduce machining costs and improve surface roughness. Alam et al. [33] combined RSM and genetic algorithms to optimize high-speed end milling cutting parameters. Lestari et al. [34] used RSM to optimize parameters in the manufacturing of ankle–foot prostheses. Vavruska et al. [35] developed an automatic feed rate optimization algorithm to achieve a constant feed for each tooth in the tool path.

Therefore, to determine the critical cutting parameters for effective chip formation in the micro-milling of curved thin-walled parts, the plastic behavior of materials and the strain-softening effect at the mesoscale were considered. A prediction model for the micro-milling of curved micro-thin walls in titanium alloy was developed to elucidate the influence mechanisms of process parameter variations on machining deformation. On this basis, a response surface experimental design was carried out to study the impact of different machining parameters on micro-thin-wall deformation. Using the improved NSGA-III algorithm combined with the entropy weight–TOPSIS method, the multi-objective optimization of milling parameters was performed. The optimized milling parameters were validated through milling experiments, verifying the effectiveness of the improved multi-objective optimization method.

## 2. Finite Element Analysis of Mesoscale Micro-Milling of a Curved Thin Wall

The influence of milling parameters on the dimensional accuracy of workpieces significantly differs between conventional and mesoscale machining. To determine the critical cutting parameters for chip formation in thin-walled micro-milling, which then serve as the foundation for the subsequent response-surface-based experimental design, it is essential to establish a finite element model for mesoscale thin-walled micro-milling. This model facilitates the study of the milling parameters that enable effective material removal in thin-walled structures.

### 2.1. Finite Element Modeling

Accurately predicting mesoscale milling deformation through simulations requires that the material constitutive model effectively captures the plastic deformation behavior of the workpiece material under large strains and high strain rates at the mesoscale. Traditional Johnson–Cook constitutive models, typically used for macroscale nonlinear deformation, fail to accurately represent mechanical behaviors at the mesoscale. Therefore, this study incorporated the scale effects in Formula (1) of mesoscale cutting and the strain-softening effects of the workpiece material in Formula (2) to develop a plastic constitutive model tailored to the thin-walled micro-milling of Ti6Al4V alloy.

(1) $$\sigma_{g}=\sigma_{mjc}\sqrt{1+{\left(\frac{18{\bar{r}}^{2}G^{2}b_{u}}{\sigma_{mjc}L_{SG}}\right)}^{s}}$$

(2) $$\sigma_{mjc}=(A+B\varepsilon^{n}(\frac{1}{\exp\left(\varepsilon^{c}\right)}))(1+C\ln\frac{\dot{\varepsilon }}{{\dot{\varepsilon }}_{0}})(1-{\left(\frac{T-T_{r}}{T_{m}-T_{r}}\right)}^{m})(D_{jc}+(1-D_{jc})\tanh(\frac{1}{{\left(\varepsilon +S\right)}^{d}}))$$

where $D_{jc}=1-{\left(T/T_{m}\right)}^{e}$, $S={\left(T/T_{m}\right)}^{f}$, A is the quasi-static yield strength, B is the strain hardening coefficient, n is the strain hardening exponent, C is the strain rate sensitivity coefficient, m is the thermal softening exponent, ε is the equivalent plastic strain, $\dot{\varepsilon }$ is the strain rate, ${\dot{\varepsilon }}_{0}$ is the reference strain rate, $T_{m}$ is the melting point, $T_{r}$ is the ambient temperature, T is the workpiece temperature, G is the shear modulus, $b_{u}$ is the amplitude of the Burgers vector, $L_{SG}$ is the characteristic length of the material within the Bing, and c,d,e,f are material constants. The Johnson–Cook constitutive parameters and thermodynamic properties of Ti6Al4V titanium alloy are listed in Table 1 and Table 2, respectively.

In this study, the Johnson–Cook damage model in Formula (3) was employed to describe the damage onset of Ti6Al4V alloy during mesoscale micro-milling.

(3) $${\bar{\varepsilon }}_{d}^{pl}=\left(D_{1}+D_{2}\exp\left(D_{3}\frac{P}{\bar{\sigma }}\right)\right)\left(1+D_{4}\ln\frac{\dot{\varepsilon }}{{\dot{\varepsilon }}_{0}}\right)\cdot \left(1+D_{5}\frac{T-T_{r}}{T_{m}-T_{r}}\right)$$

where ${\bar{\varepsilon }}_{d}^{pl}$ is the equivalent plastic strain at the onset of damage, P is hydrostatic pressure, $\bar{\sigma }$ is von Mises effective stress, and *D*_1_*~D*_5_ are material constants. The Johnson–Cook damage model parameters of the Ti6Al4V material are shown in Table 3.

Once material damage is initiated, the plastic constitutive model can no longer accurately capture the post-damage plastic deformation behavior. Therefore, the effective plastic displacement, as defined by Formula (4), is utilized to describe the damage evolution of a material during the mesoscale milling process.

(4) $$\dot{d}=\frac{L{\dot{\varepsilon }}^{pl}}{{\bar{u}}_{f}^{pl}}=\frac{{\dot{u}}^{pl}}{{\bar{u}}_{f}^{pl}}$$

where $\dot{d}$ represents the damage variable, L is the characteristic length of the element, ${\dot{u}}^{pl}$ is the equivalent plastic displacement during the material failure process, and ${\bar{u}}_{f}^{pl}$ is the equivalent plastic displacement at complete material failure. When ${\dot{u}}^{pl}$ = ${\bar{u}}_{f}^{pl}$, the element is considered to have fully failed.

**Figure 1:**
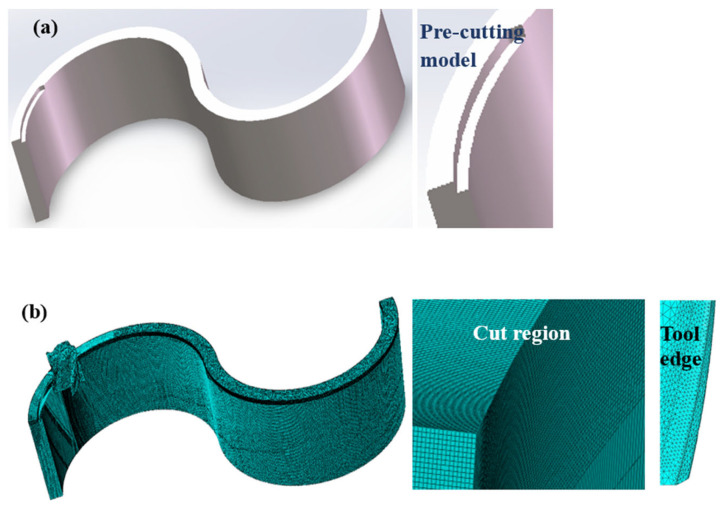
Micro-milling of curved thin wall: (**a**) pre-cutting geometry; (**b**) finite element model.

To accelerate a milling simulation, a pre-cutting method is employed, as shown in Figure 1a. The curved thin-walled part is meshed using hexahedral elements, with local mesh refinement applied to the milling region, while transition meshes are used in other regions to reduce computational costs. The milling cutter is meshed using tetrahedral elements and the cutting edges are further refined to enhance the simulation accuracy. Full constraints are imposed on the bottom of the thin-walled part, while the milling cutter is assigned a specific rotational speed and moves along a predefined trajectory.

A modified Coulomb model is used for the friction contact of the tool–chip and tool–workpiece. The friction contact zone is divided into adhesive and sliding friction zones. The frictional stress $\tau_{f}$ at the contact surface is calculated as shown in Formula (5).

(5) $$\left\{\begin{array}{l} \tau_{f}=\mu \sigma_{n},\;\tau_{f}<\tau_{\max}\;(\mathrm{Slip}\;\mathrm{friction}\;\mathrm{zone})\\ \tau_{f}=\tau_{\max},\;\tau_{f}\geq \tau_{\max}\;(\mathrm{Adhesive}\;\mathrm{friction}\;\mathrm{zone}) \end{array}\right.$$

where μ is the friction coefficient, $\sigma_{n}$ is the normal stress on the tool–chip contact surface, and $\tau_{\max}$ is the critical shear yield strength of workpiece material.

### 2.2. Analysis of Results

**Figure 2:**
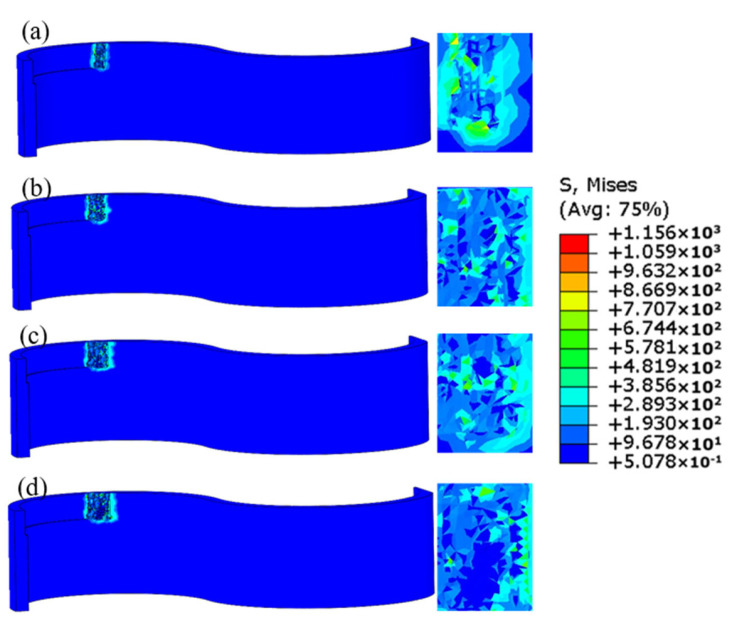
Equivalent stress distribution of a curved thin-walled structure during micro-milling process: (**a**) t = 0.0075 s; (**b**) t = 0.015 s; (**c**) t = 0.0225 s; (**d**) t = 0.03 s.

The finite element simulation’s corresponding stress distribution cloud diagram at various intervals is shown in Figure 2. It was evident that there was significant node deformation at the start of milling, which is detrimental to processing. However, the stress was focused at the front end of the contact when the thin-walled sections were gradually milled by the milling cutter. The workpiece experienced irreversible deformation when its highest stress was above the material’s yield limit. The unit grid was removed upon reaching the material failure criteria. Optimal chips could be generated by these cutting process parameters to serve as a benchmark for the subsequent experimental development.

Figure 2 shows the equivalent stress distribution of the curved thin-walled structure during the micro-milling process at a spindle speed of 14,000 r/min, a feed per tooth of 3 µm/z, a radial depth of cut of 60 µm, and an axial depth of cut of 300 µm. Under these milling parameters, chip formation occurred, resulting in effective material removal. At the beginning of the cutting process, a large area of high-stress concentration was formed in the region to be machined. The significant deformation at this stage was detrimental to machining. However, as the tool gradually cut into the thin-walled part, the stress concentration was limited to the leading edge of the contact area, and no excessive stress concentration was observed. When the stress on the workpiece exceeded the material’s yield strength, irreversible deformation occurred and chip formation began once the plastic deformation reached the material failure criterion. Therefore, under these micro-milling parameters, effective chip formation was achieved, providing a reference for the subsequent response surface experimental design.

**Figure 3:**
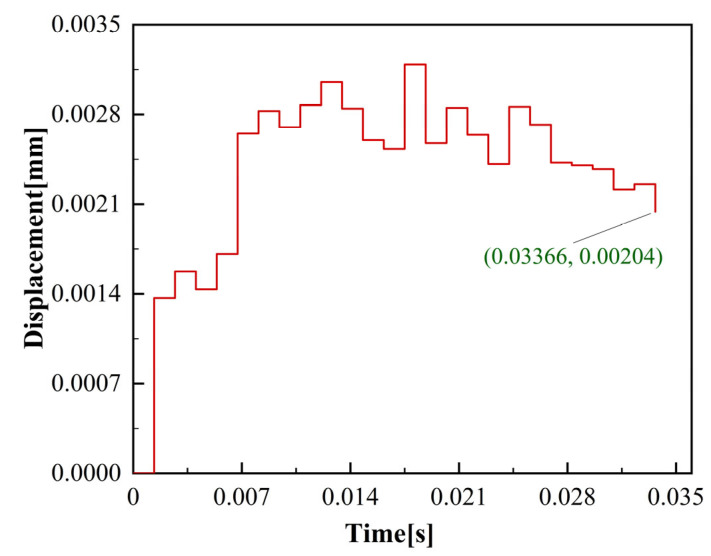
Deformation measurement of the thin-wall top fixed point.

The evolution of deformation at the top of the thin-walled structure during mesoscale micro-milling is shown in Figure 3. As the micro-milling tool gradually cut in, the deformation of the curved thin-walled structure gradually increased. As the tool cut out, the elastic deformation of the thin wall recovered and the amount of deformation decreased. At this point, the unmachined section of the thin wall exhibited relatively high stiffness, resulting in only the minor unidirectional deformation of the thin wall.

## 3. Experimental Study of Milling Deformation Based on Response Surface Methodology

### 3.1. Experimental Design Based on Response Surface Methodology

**Figure 4:**
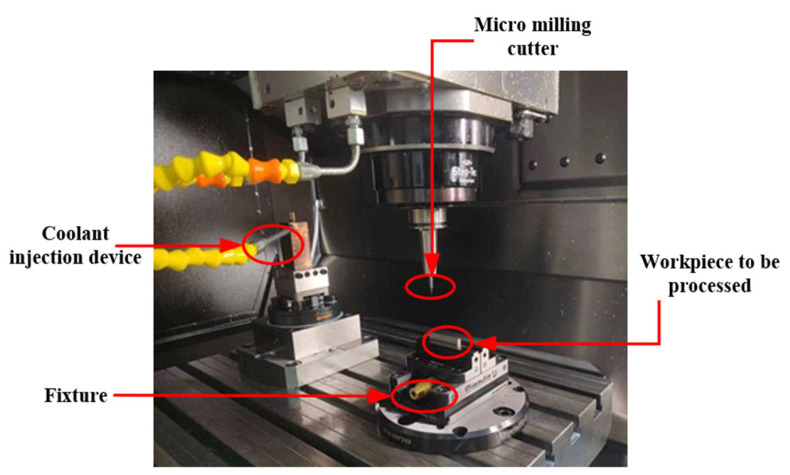
Micro-milling device.

The experimental milling operations were conducted using a GF MIKRON HSM500 high-speed machining center with a repetitive positioning accuracy of 1 μm. The workpiece was clamped using precision fixtures, and the micro-milling experimental setup for the curved thin-walled structure is shown in Figure 4. The cutting tool used was a 4-flute end mill NS MHRH430R with a diameter of 0.5 mm. The workpiece size was 40 mm × 10 mm × 5 mm. The final finishing operations targeted curved thin-walled parts with a wall thickness of 80 μm and a height of 600 μm, and twelve curved thin-walled features were machined on each workpiece. The chemical composition of the workpiece material Ti6Al4V is listed in Table 4. In this experiment, the central composite design (CCD) method was used for the experimental design, and four milling parameters (spindle speed *n*, feed per tooth *f_z_*, radial depth of cut *a_e_*, and axial depth of cut *a_p_*), which have a significant impact on the dimensional accuracy of thin-walled parts, were selected as design variables. Each design variable was set at three levels, and the experimental arrangements based on RSM are shown in Table 5 with the corresponding response values recorded.

The standard deviation (*σ*) was used as a metric to reflect the variability in the measured thin-wall thickness compared with the mean value, serving as an indicator of the fluctuations in the thin-wall thickness during micro-machining. This metric provides valuable information on the variability and distribution of the wall thickness data for curved thin-walled structures. Additionally, the top surface deformation (*D*) is a critical indicator for assessing the dimensional accuracy of a curved micro-thin wall during milling.

**Figure 5:**
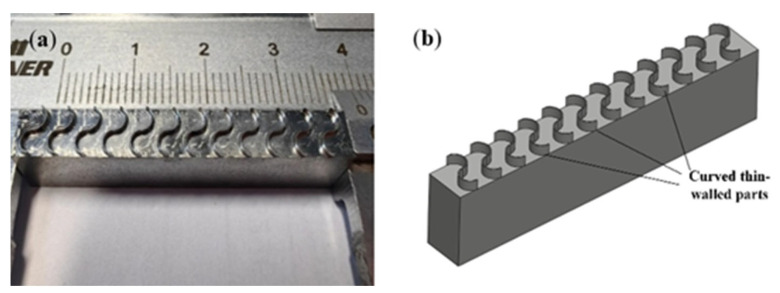
Finishing of curved thin-wall structure: (**a**) machined structure; (**b**) structure design diagram.

**Figure 6:**
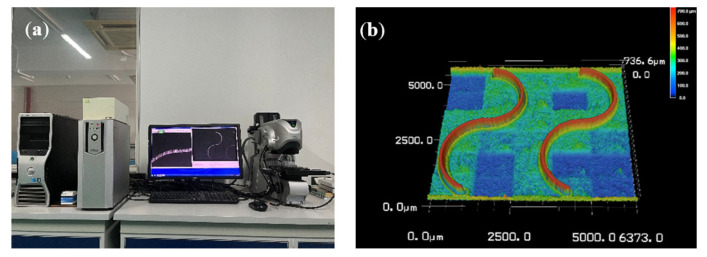
(**a**) Top surface profile thickness measurement; (**b**) 3D morphology of curved thin wall.

The actual finished surface and the designed curved thin-walled structure are shown in Figure 5. Based on the milling parameters determined from the simulation analysis of the micro-milling of a curved thin-wall part, 24 groups of micro-milling experiments were designed using RSM. The top surface deformation was measured using a Keyence VK-X200K (Osaka, Japan) laser confocal microscope, which had a resolution of 0.001 μm. Figure 6 shows the measured values of the top surface profile thickness after milling and the 3D morphology of the curved thin-walled structure.

### 3.2. Regression Equations and Analysis of Variance

To establish the functional relationship between the deformation value of Ti6Al4V titanium alloy after high-speed milling and the cutting parameters, a second-order equation was used to fit the response surface. The resulting quadratic function model is expressed as follows:

(6) $$Y=\beta_{0}+\sum \beta_{i}x_{i}+\sum \beta_{ii}x_{i}^{2}+\sum \sum \beta_{ij}x_{i}x_{j}+\theta$$

where *Y* is the model response, $\beta_{i}$ and $\beta_{0}$ are regression coefficients, $x_{i}$ represents the coefficient to be determined, and θ is the estimated error. The mathematical relationship between the top surface deformation amount (*D*) and the milling parameters (spindle speed *n*, feed per tooth *f_z_*, radial depth of cut *a_e_*, and axial depth of cut *a_p_*) is given by Formula (7).

(7) $$\begin{array}{l} D=39.22274-0.000724\times n+1.27697\times fz-1.28447\times ae+0.049547\times ap\\ +0.000072\times nf_{z}-6.91558\times 10^{-6}na_{e}-5.50802\times 10^{-7}\times na_{p}-0.016384\times f_{z}a_{e}+0.004679\\ \times f_{z}a_{p}+0.000319\times a_{e}a_{p}+2.10525\times 10^{-8}\times n^{2}-0.411630\times f_{z}^{2}+0.016343\times a_{e}^{2}\\ -0.000193\times a_{p}^{2} \end{array}$$

**Figure 7:**
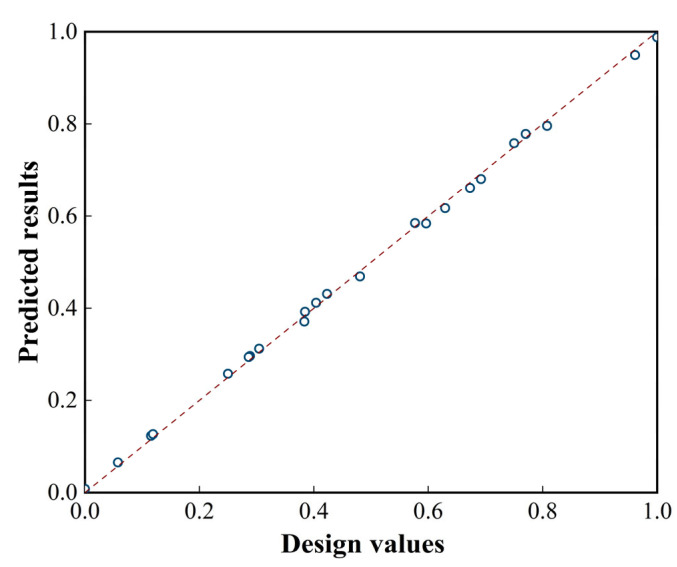
Distribution of predicted results and design values.

To verify the reliability of the regression model and the significance of the key process parameters, an analysis of variance (ANOVA) was conducted. The *p*-value for the overall model was less than 0.0001, indicating a significant regression equation between each design factor and response value. Additionally, the *p*-values for the factors *a_p_*, *nf_z_*, *na_e_*, *na_p_*, *f_z_a_p_*, *a_e_*^2^, *n*^2^, and *a_p_*^2^ were all less than 0.05, indicating statistical significance. A comparison of the predicted results of the design values is shown in Figure 7.

### 3.3. Effects of Milling Parameters on Machining Deformation

**Figure 8:**
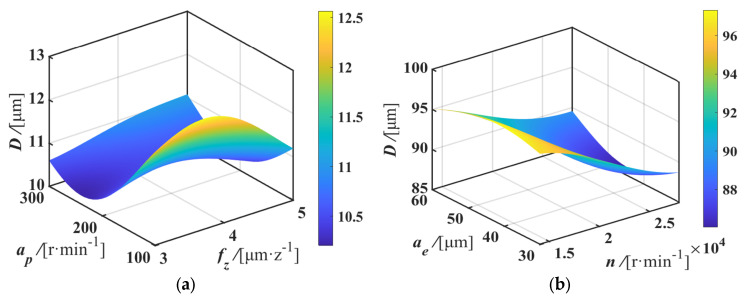

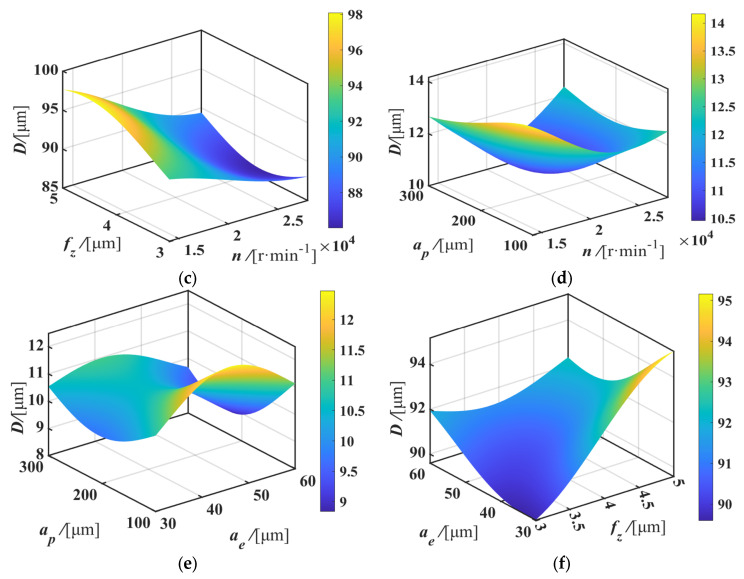
Effects of milling parameters on the machining deformation *D* of curved micro-thin-walled structures: (**a**) *f_z_* and *a_p_*; (**b**) *n* and *a_e_*; (**c**) *f_z_* and *n*; (**d**) *n* and *a_p_*; (**e**) *a_e_* and *a_p_*; (**f**) *a_e_* and *f_z_*.

As shown in Figure 8, the response surface provided a more intuitive depiction of the influence of each milling parameter on the machining deformation of the micro-thin-walled structure. It was observed that among all the milling parameters, the interactions between the parameters had a significant impact on top surface deformation. Specifically, a higher spindle speed (20,000~25,000 r/min) could mitigate the negative impact of a large feed per tooth on top surface deformation, while a higher axial depth of cut (200~300 μm) tended to exacerbate this negative effect. Therefore, when selecting large radial and axial depths of cut, it is recommended that higher spindle speeds are used to reduce the adverse effects of a high material removal rate, thereby achieving smaller deformation values. Consequently, using a larger axial depth of cut and a smaller radial depth of cut leads to greater deformation, and higher spindle speeds (20,000~25,000 r/min) should be employed to minimize deformation.

## 4. Optimization of Micro-Milling Process Based on Machining Deformation Control

### 4.1. Multi-Objective Optimization Model

To reduce deformation in the micro-milling of thin-walled structures, a mathematical representation was developed to minimize the deviation between the measured thickness of the machined thin-walled part and the design value, which was set as one of the minimization objectives. Additionally, considering the consistency differences in the thickness at different positions along the curved thin-walled structure, the standard deviation of the thickness errors within the same group was selected as another minimization objective. Given the layered milling process, the constraint condition was that the height of the thin wall must be an integer multiple of the axial depth of cut. A multi-objective optimization model was constructed using the spindle speed *n*, feed per tooth *f_z_*, radial depth of cut, and axial depth of cut as design variables, as shown in Formula (8).

(8) $$\begin{array}{l} find:x_{1},x_{2},x_{3},x_{4}\\ min:\left\{\begin{array}{l} f_{1}(x_{1},x_{2},x_{3},x_{4})=\left|D-D_{design}\right|\\ f_{2}(x_{1},x_{2},x_{3},x_{4})=\left|\sigma \right| \end{array}\right.\\ s.t.:\left\{\begin{array}{c} 14,000\leq x_{1}\leq 28,000\\ 3\leq x_{2}\leq 5\\ 30\leq x_{3}\leq 60\\ 100\leq x_{4}\leq 300\\ x_{4}=600\times \frac{1}{n},n\in N^{*} \end{array}\right. \end{array}$$

where *D_design_* is the design value of the curved thin-walled structure and *x*_1_*~x*_4_ are the milling parameters to be determined.

### 4.2. Improved NSGA-III Optimization Based on TOPSIS Evaluation

NSGA-III is a reference-point-based multi-objective optimization algorithm. By introducing the reference point mechanism, NSGA- III enhances the selection mechanism of NSGA-II, which is based on the crowding distance, improving both the convergence and diversity of the population while reducing the time complexity and providing better convergence and distribution performance under complex constraints. However, as the crossover and mutation operators in the NSGA-III algorithm use fixed rates, the algorithm can suffer from premature convergence and poor search capabilities. If the crossover probability is too high, new individuals are rapidly generated but the genetic model is easily disrupted. Conversely, if the crossover probability is too low, the search speed is slow. Similarly, if the mutation rate is too high, high-fit individuals are destroyed, slowing down the evolution process. If the mutation rate is too low, most individuals proceed directly to the next generation, decreasing the search capability.

**Figure 9:**
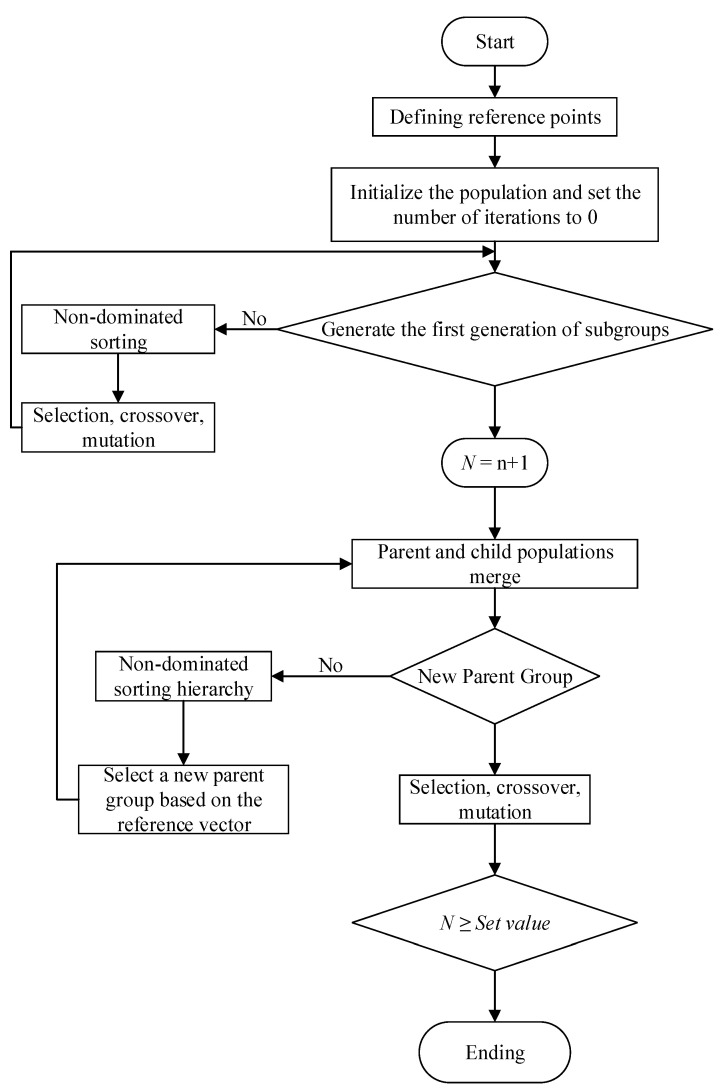
Optimization algorithm solution process.

To improve the convergence speed and search capability of the NSGA-III algorithm, the crossover and mutation rates were adaptively modified as Formulas (9) and (10).

(9) $$P_{c}^{dynamic}=\varepsilon -\frac{2e^{-n/N}}{1+e^{-n/N}}\times P_{c}$$

(10) $$P_{m}^{dynamic}=\frac{{2e}^{-n/N}}{1+e^{-n/N}}\times P_{m}$$

where $P_{c}$ is the fixed crossover rate, $P_{m}$ is the fixed mutation rate, *n* is the current number of generation, and *N* is the maximum number of generation. The algorithm principle of the improved NSGA-III is illustrated in Figure 9.

**Figure 10:**
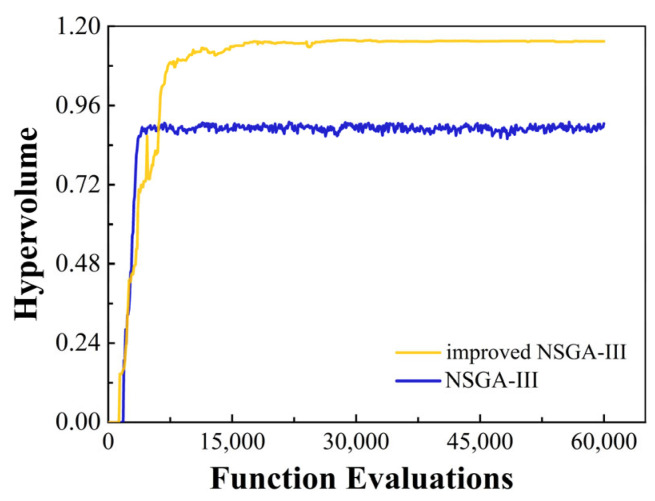
Hypervolume indicators of the obtained solution sets using standard NSGA-III and an improved NSGA-III.

The hypervolume (*HV*) indicator is used to measure the volume of the objective space dominated by at least one solution in a non-dominated solution set. *X* denotes the non-dominated solution set obtained by the algorithm and *P* represents the reference point corresponding with the true Pareto front, typically a vector formed by the maximum values of each objective. The hypervolume of the non-dominated solution set relative to the true Pareto front is then calculated as follows:

(11) $$HV(X,P)=\overset{X}{\underset{x\in X}{\cup }}\nu (x,P)$$

where HV(X,P) represents the hypervolume formed between the solution *x* in the non-dominant solution set *X* and the reference point *P*, which can evaluate the convergence and diversity of the dominant solution set without the need for a true Pareto front. Figure 10 shows the hypervolume indicators of the solution sets calculated by NSGA-III and the improved NSGA-III, respectively.

**Figure 11:**
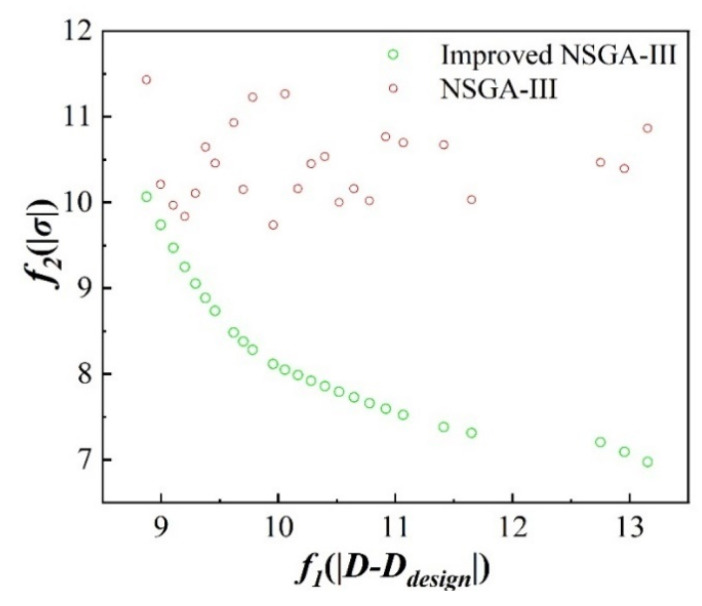
Comparison of Pareto optimal solution sets obtained by NSGA-III and improved NSGA-III algorithms.

The maximum number of iterations was set to 600 and the population size was 400. The multi-objective optimization of deformation control in the high-speed milling of a micro-thin-walled structure was carried out to obtain the Pareto front optimal solution set. The Pareto optimal solution sets obtained by NSGA-III and the improved NSGA-III algorithms are compared in Figure 11. The improved NSGA-III consistently produced a more optimal trade-off between the two objectives, with the solutions clustered closer to the lower values of both *f*_1_ and *f*_2_. Specifically, the improved NSGA-III algorithm reduced both the deviation from the design thickness and the variability (standard deviation) in thickness, achieving better convergence and diversity in the solution set. The improved NSGA-III found solutions that minimized both the thickness deviation and the standard deviation of thickness more effectively than the standard NSGA-III algorithm.

The Pareto front optimal solution sets obtained by the improved NSGA-III algorithm contained 24 groups of parameter combinations, as listed in Table 6. The TOPSIS method was used to perform optimal decision-making on the obtained Pareto front optimal solution set. TOPSIS is a comprehensive evaluation method that can fully utilize original data and accurately reflect the differences between evaluation schemes. The improved NSGA-III optimization based on the TOPSIS evaluation had no strict restrictions on data distribution or the sample size, and the calculation process was simple and straightforward.

The final TOPSIS optimal solution decision results according to the degree of proximity between the current Pareto front optimal solution set and the idealized optimal solution are shown in Table 6. The 10th group of solutions was the optimal solution to the milling parameter optimization problem, and had the following milling parameters: spindle speed *n* = 23,306.78 r/min, feed per tooth *f_z_* = 3.08 μm/z, radial depth of cut *a_e_* = 41.62 μm, and axial depth of cut *a_p_* = 300.00 μm.

### 4.3. Experimental Validation of Optimal Milling Parameters

**Figure 12:**
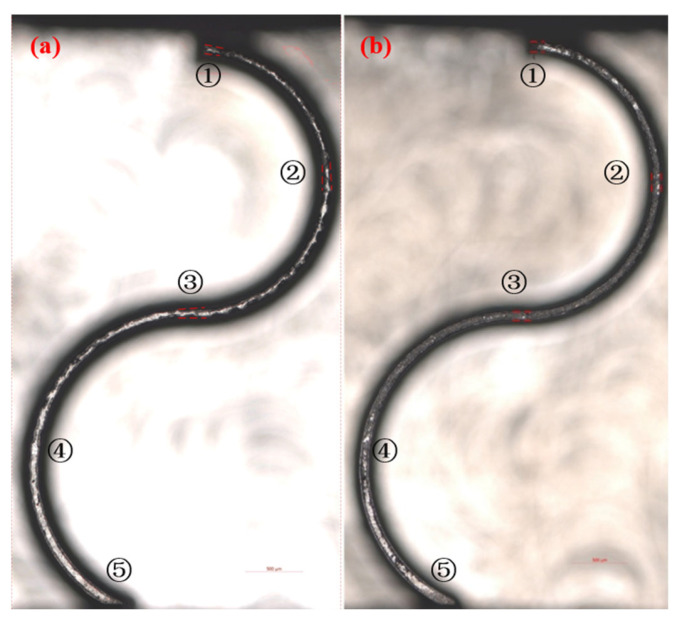
Comparison of milled curved thin-walled structures using the optimal milling parameters obtained by (**a**) standard NSGA-III and (**b**) improved NSGA-III algorithms. (1~5 represent the five positions of the deformation measurement).

**Figure 13:**
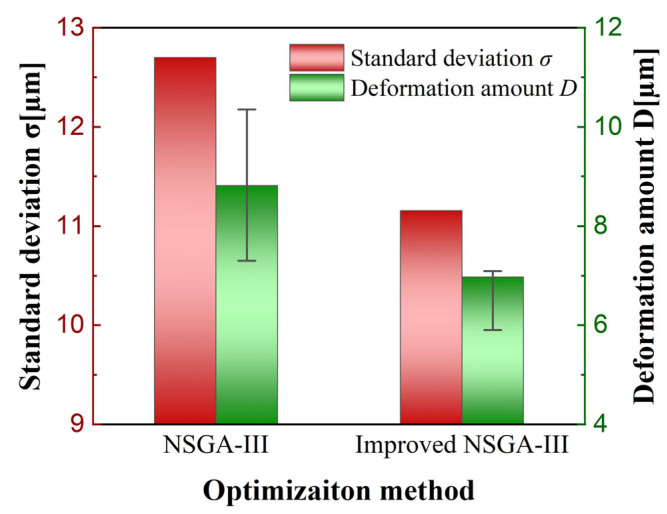
Statistical analysis of standard deviation and thickness deformation error before and after optimization.

Based on the optimal process parameter combination obtained from the standard NSGA-III and the improved NSGA-III based on the TOPSIS evaluation, micro-milling validation experiments were conducted on the curved micro-thin-walled structures. A comparison of milled curved thin-walled structures using the optimal milling parameters obtained by the standard NSGA-III and the improved NSGA-III algorithms is shown in Figure 12. The results showed that the optimized milling parameters obtained by the improved NSGA-III algorithm achieved good machining accuracy and there was no obvious deformation of the curved thin-walled structure. Compared with the optimization scheme of the standard NSGA-III, the optimization parameters based on the improved NSGA-III algorithm reduced the deformation of the thin-wall top surface by 20.894% and the standard deviation of the thin-wall thickness by 12.164%. The comparison of the standard deviation of the thin-wall thickness and the deformation of the thin-wall top surface based on the standard NSGA-III and the improved NSGA-III algorithms is shown in Figure 13, which confirms the effectiveness of the improved NSGA-III optimization based on the TOPSIS evaluation in improving the machining quality of curved thin-walled structures.

## 5. Conclusions

In this study, a mesoscale micro-milling finite element model of curved thin-walled Ti6Al4V structures was established to analyze stress-induced deformation at the top of a micro-thin wall during milling and to determine the critical milling parameters for chip formation, which served as the basis for the response surface experimental design. The analysis of variance revealed that high spindle speeds (20,000~25,000 r/min) could mitigate the adverse effects of a large feed per tooth, while larger axial depths of cut exacerbated top surface deformation. The influence of various milling parameters on the deformation of the mesoscale thin wall was then investigated. By integrating an improved NSGA-III algorithm with the entropy-weighted TOPSIS method, a dynamic multi-fobjective optimization strategy for process parameters in the micro-milling of curved thin-walled structures was achieved, reducing both the deviation from the design thickness and the variability in thin-wall thickness and demonstrating the effectiveness of the proposed approach in controlling micro-thin-wall deformation during a mesoscale milling process. The micro-milling experimental validation using the optimal milling parameters obtained by the improved NSGA-III algorithm confirmed that the deformation of the thin-wall top surface was reduced by 20.894% and the standard deviation of the thin-wall thickness decreased by 12.164% compared with the standard NSGA-III algorithm. This validated the robustness of the proposed multi-objective optimization strategy for improving the machining quality of curved thin-walled structures.

## Figures and Tables

**Table 1:** Johnson–Cook constitutive parameters of Ti6Al4V alloy.

*A*	*B*	*C*	*n*	*m*	${\dot{\varepsilon }}_{0}$
782.7	498.4	0.028	0.28	1	10^−2^

**Table 2:** Material properties of the Ti6Al4V workpiece.

Material Parameter	Ti6Al4V
Elastic modulus [GPa]	113
Poisson ratio	0.342
Density [kg/m^3^]	4430
Specific heat capacity [J/(kg·°C)]	546
Thermal conductivity [W/(m·°C)]	7.0
Melting point [°C]	1680

**Table 3:** Johnson–Cook damage model parameters of Ti6Al4V alloy.

*D*_1_	*D*_2_	*D*_3_	*D*_4_	*D*_5_
−0.09	0.27	−0.50	0.014	3.87

**Table 4:** Chemical composition of the Ti6Al4V workpiece.

Chemical Elements	Fe	C	N	H	O	AL	V	Ti
Contents/%	0.3	0.1	0.05	0.015	0.2	6.07	4.12	Margin

**Table 5:** Arrangement and response values of RSM experiment.

Standard	Run	*n*	*f_z_*	*a_e_*	*a_p_*	*σ*	*D*
Sequence	Sequence	r/min	μm/z	μm	μm	μm	μm
13	1	14,000	3	60	300	10.406	13.144
14	2	28,000	3	60	300	11.474	13.859
24	3	21,000	4	45	300	10.978	7.281
21	4	21,000	4	30	200	9.651	12.764
23	5	21,000	4	45	100	12.533	10.571
11	6	14,000	5	30	300	12.886	14.518
17	7	14,000	4	45	200	13.436	17.368
19	8	21,000	3	45	200	10.342	10.337
3	9	14,000	5	30	100	12.163	16.930
8	10	28,000	5	60	100	12.158	15.175
16	11	28,000	5	60	300	12.701	8.816
12	12	28,000	5	30	300	12.206	13.640
7	13	14,000	5	60	100	14.183	14.748
6	14	28,000	3	60	100	13.290	9.435
2	15	28,000	3	30	100	12.724	7.322
18	16	28,000	4	45	200	11.983	5.965
10	17	28,000	3	30	300	13.636	9.254
22	18	21,000	4	60	200	8.897	11.448
5	19	14,000	3	60	100	11.591	9.228
9	20	14,000	3	30	300	12.277	10.790
4	21	28,000	5	30	100	11.773	10.351
1	22	14,000	3	30	100	11.026	6.623
20	23	21,000	5	45	200	10.230	12.544
15	24	14,000	5	60	300	10.659	11.009

**Table 6:** Pareto front optimal solution set and TOPSIS optimal solution decision-making.

Serial	*n*	*f_z_*	*a_e_*	*a_p_*	σ	*D*	Optimal
Number	r/min	μm/z	μm	μm	μm	μm	Sorting
0	14,000.02	4.93	33.11	300.00	9.201	9.248	24
1	16,235.92	5.00	40.33	300.00	10.917	7.593	21
2	14,000.06	4.86	32.57	300.00	9.104	9.472	20
3	14,747.14	5.00	39.80	300.00	10.397	7.859	16
4	14,003.84	5.00	39.26	300.00	10.057	8.047	12
5	14,000.08	5.00	37.26	300.00	9.781	8.282	11
6	22,244.39	3.32	41.43	300.00	12.750	7.206	6
7	15,789.57	5.00	40.20	300.00	10.777	7.661	7
8	14,177.72	5.00	39.59	300.00	10.167	7.985	5
9	14,000.01	5.00	36.66	300.00	9.699	8.378	4
10	23,306.78	3.08	41.62	300.00	11.452	6.974	1
11	16,588.24	5.00	40.81	300.00	11.065	7.524	2
12	14,000.01	5.00	37.89	300.00	9.866	8.194	3
13	17,834.47	5.00	41.49	300.00	11.413	7.382	8
14	14,001.94	5.00	35.46	300.00	9.541	8.604	9
15	18,899.86	5.00	42.12	300.00	11.649	7.314	10
16	22,985.49	3.00	42.74	300.00	13.391	6.853	14
17	17,157.71	5.00	41.06	300.00	11.228	7.453	15
18	14,000.02	5.00	36.06	300.00	9.620	8.485	13
19	14,000.00	5.00	34.86	300.00	9.460	8.736	17
20	14,000.06	4.78	30.93	300.00	8.876	10.067	18
21	14,000.00	4.58	30.00	300.00	8.735	10.544	19
22	14,000.00	4.96	33.71	300.00	9.292	9.055	22
23	14,000.14	4.82	31.81	300.00	8.996	9.739	23

## Data Availability

The original contributions presented in the study are included in the article; further inquiries can be directed to the corresponding author.
